# Development and initial psychometric properties of the Research Complexity Index

**DOI:** 10.1017/cts.2024.534

**Published:** 2024-05-09

**Authors:** Allison A. Norful, Bernadette Capili, Christine Kovner, Olga F. Jarrín, Laura Viera, Scott McIntosh, Jacqueline Attia, Bridget Adams, Kitt Swartz, Ashley Brown, Margaret Barton-Burke

**Affiliations:** 1 Columbia University School of Nursing, New York, NY, USA; 2 Rockefeller University, New York, NY, USA; 3 New York University Rory Meyers College of Nursing, New York, NY, USA; 4 Rutgers The State University of New Jersey, New Brunswick, NJ, USA; 5 University of North Carolina, Chapel Hill, NC, USA; 6 University of Rochester Medical Center–CLIC, Rochester, NY, USA; 7 Oregon Health & Science University, Portland, OR, USA; 8 Memorial Sloan Kettering Cancer Center, New York, NY, USA

**Keywords:** Clinical research, instrumentation, psychometric, research design, workload

## Abstract

**Objective:**

Research study complexity refers to variables that contribute to the difficulty of a clinical trial or study. This includes variables such as intervention type, design, sample, and data management. High complexity often requires more resources, advanced planning, and specialized expertise to execute studies effectively. However, there are limited instruments that scale study complexity across research designs. The purpose of this study was to develop and establish initial psychometric properties of an instrument that scales research study complexity.

**Methods:**

Technical and grammatical principles were followed to produce clear, concise items using language familiar to researchers. Items underwent face, content, and cognitive validity testing through quantitative surveys and qualitative interviews. Content validity indices were calculated, and iterative scale revision was performed. The instrument underwent pilot testing using 2 exemplar protocols, asking participants (*n* = 31) to score 25 items (e.g., study arms, data collection procedures).

**Results:**

The instrument (Research Complexity Index) demonstrated face, content, and cognitive validity. Item mean and standard deviation ranged from 1.0 to 2.75 (Protocol 1) and 1.31 to 2.86 (Protocol 2). Corrected item-total correlations ranged from .030 to .618. Eight elements appear to be under correlated to other elements. Cronbach’s alpha was 0.586 (Protocol 1) and 0.764 (Protocol 2). Inter-rater reliability was fair (kappa = 0.338).

**Conclusion:**

Initial pilot testing demonstrates face, content, and cognitive validity, moderate internal consistency reliability and fair inter-rater reliability. Further refinement of the instrument may increase reliability thus providing a comprehensive method to assess study complexity and related resource quantification (e.g., staffing requirements).

## Introduction

The development and implementation of clinical research studies are challenging for investigators [1,2]. Barriers may include complex regulatory requirements, restrictive eligibility criteria, specific study timelines, and limited funding to support a study. To overcome these barriers and ensure quality and integrity [1,3], studies must have sufficiently trained personnel to conduct the research. Specifically, clinical studies require appropriate staffing to support screening for establishing trial eligibility, participant recruitment and retention, obtaining informed consent, ensuring fidelity to treatment (e.g., study maintenance and adherence), and complying with adverse event (AE) reporting and follow-up. Despite the need for appropriate staffing, few quantitative models inform staffing needs to ensure the study’s safe conduct, achievement of study goals, and budget adherence. Further, variability across protocol complexity often presents challenges to planning appropriate allocation of resources [4].

A literature review confirmed that methods to estimate clinical research workload and study complexity across all study design types are scant. The majority of literature describes complexity in the context of clinical trials and is often isolated to oncology or pharmaceutical trials [5–7]. Instruments specific to oncology research exist and include the *Ontario Protocol Assessment Level*^*1*^, the *Wichita Community Clinical-Trial Oncology Protocol Acuity Tool*^*3*^, and the *NCI Trial Complexity Elements & Scoring Model* [8]. These instruments do not differentiate between inpatient or outpatient settings, nor do they differentiate by cohort or credentials of the research staff (e.g., technician, clinical research nurse [RN], non-RN coordinator) who implement the research activities. Also, definitions for workload, study complexity, and methods to evaluate existing instruments’ reliability and validity are not described in detail.

Clinical research staff, often RNs, are responsible for managing complex teams and workflow of clinical research studies including (1) managing relationships with a variety of staff; (2) protocol review, logistics, staff adjustments, and budget; (3) protocol approval process including managing scientific and institutional review board committee meetings, reviews of protocol instruments, sponsor communication, and staff education; (4) research participant prescreening, protocol visit management, AEs, source documentation, invoicing, and query resolution; and (5) sponsor correspondence including AE reporting, monitoring sponsor visits, and study startup and close-out visits. These aspects of clinical research staff responsibilities must be accounted for when assessing study workload and complexity. Such metrics can provide research teams with an objective method to quantify the activities associated with clinical research studies based on factors contributing to workload. Thus, the purpose of this multi-phase study was to develop an instrument that may be used across all study design types to scale research complexity. This article describes the first two phases of the development of the Research Complexity Index (RCI): (1) identifying core elements of clinical research studies and (2) developing initial items to scale each element and evaluating the tool’s initial psychometric properties.

## Materials and methods

### Phase 1. Item development; content, face, and cognitive validity testing

#### Item development

In preliminary work, we conducted a literature review with content analysis to identify conceptual dimensions, definitions, and any existing instruments that scale research study complexity. The research team classified the content guided by the Donabedian model [9] – a process involving *Structure* (e.g., environment, personnel, resources), *Processes* (e.g., procedures), and *Outcomes (e.g., study deliverables, dissemination goals).* See Table 1. Through iterative revisions, we established a working definition of the construct of research complexity: *Clinical research study complexity* is defined as the elements that contribute to the intricacy and difficulty of a clinical study. It includes elements such as the nature of the intervention (e.g., novel drugs, complex procedures), the design of the study (e.g., randomized controlled trials, multi-site studies), the study population (e.g., rare diseases, multiple comorbidities), regulatory requirements, and data management needs. A complex study typically requires advanced planning and specialized expertise to manage and execute the study effectively.

**Table 1.** Proposed dimensions of research study and trial complexity (adapted from Donabedian’s model)

Structure			Process		Outcomes
*Environment*	*Personnel*	*Resources*	*Procedures*	*Team-based processes*	*Study outcomes*
• Ample physical space in primary institution to conduct study procedures	• Research Team Size/Composition	• Required equipment & supplies to carry out procedures	• Study procedures	• Delineated roles and responsibilities for each study team member	• Novel evidence produced that warrants investigation and future research
			○ recruitment and enrollment		
	• Research team experience level	• Sufficient Source of Funding	○ sample size		
• Institutional and Stakeholder Support				• Measures of accountability for task completion	• Plan for feedback and evaluation of methodologic success needed for subsequent study planning
	• Proposed investigator effort or allocation of time to complete study		○ study arms		
			○ randomization steps		
• Access to external environments required to conduct study procedures (if applicable)	• Access to support staff to carry out study procedures		○ intervention		
			○ study duration	• Conflict resolution	
			○ data collection		
			○ follow-up		
			○ personnel roles		
			• Feasibility of timeline for study completion		

Next, we cross-checked our elements of research complexity to the existing instrument, National Cancer Institute (NCI) Trial Complexity Elements and Scoring Model. This NCI instrument has 10 Items to assess research complexity. After iterative discussion and review of our content analysis, we added an additional 15 items and renamed some items to better capture the details of our applied theoretical model. Table 2 displays the match between the elements of the NCI Instrument with the new instrument. Guidelines for technical and grammatical principles were followed to produce and revise clear and concise items that use language familiar to clinical research professionals [10]. We then revised items and response options to be useful in various study designs rather than limiting the instrument to clinical trials. Following a review for clarity and grammar, the new items, representing each of the study’s complexity elements, underwent face, content, and cognitive validity testing [11].

**Table 2.** Alignment of new instrument with existing National Cancer Institute tool

	National Cancer Institute Tool		Research Complexity Index
**1**	Number of study arms	**1**	Study Arms
**2**	Informed consent process	**2**	Informed Consent
**3**	Registration or randomization steps	**3**	Randomization
**4**	Complexity of investigational treatment	**4**	Type of Intervention
**5**	Length of investigational treatment	**5**	Intervention Administration
**6**	Feasibility & personnel impact	**6**	Research team
		**7**	Hiring and Job Descriptions
**7**	Data collection complexity	**8**	Data Collection (Procedures)
		**9**	Data Collection (Frequency)
**8**	Follow-up requirements	**10**	Follow-up
**9**	Ancillary studies		---
**10**	Participant feasibility & enrollment	**11**	Participant Eligibility
		**12**	Access to Target Population
		**13**	Vulnerable Populations
		**14**	Expected Adverse Event/Safety
		**15**	Incentives
		**16**	IRB Prep
		**17**	Selection of Study Instruments
		**18**	Multiple PI agreements
		**19**	Physical equipment
		**20**	Budget Preparation
		**21**	Consultant Agreements
		**22**	Facilities or vendor agreement
		**23**	Compliance Reporting
		**24**	Statistical Analysis
		**25**	Dissemination

#### Data collection & analysis

In May 2022, we sent a REDCap electronic survey link via email to individuals through a random sample of institutions within the authors’ clinical research professional networks. The authors and the NCAT-funded University of Rochester Medical Center’s Center for Leading Innovation and Collaboration (CLIC) staff pretested the electronic version of the instrument for online functionality before its distribution via email. The email included a description of the project’s purpose, an anonymous survey link (content validity testing), and a request for potential participants to participate in an interview to assess face and cognitive validity. Employing a snowball technique, we also requested participants to invite colleagues to participate in the survey. CLIC staff collected and managed data using REDCap electronic data capture tools.

#### Content validity testing

Content validity ensures that the new scoring rubric and items are relevant to the content being measured [12]. Eligibility criteria to participate were self-reported: (1) five or more years’ experience in preparing, directing, or coordinating clinical studies sponsored by industry, foundation, and/or government, and (2) completed training in research, ethics, and compliance (such as offered by the Collaborative Institutional Training Initiative (CITI Program) or equivalent).

The initial pool of items was built into REDCap survey software. Participants were recruited and asked to rate each item and response options (“scoring tiers”) on a 4-point Likert scale ranging from ‘highly relevant’ (4) to ‘highly irrelevant’ (1), and, separately, from “clear, requires no revision” (4) to “unclear, consider removal” (1). To establish initial content validity, the recommended sample size is a minimum of 6 participants. A content validity index (CVI) was computed for the individual items (I-CVI) and response options (R-CVI). Indices greater than 0.8 were eligible for inclusion and further psychometric testing [12].

#### Face & cognitive validity testing

Each 1:1 interview was conducted via Zoom at a time convenient to the participant. The participant read each item aloud, interpreted what the item and response options were asking, and openly discussed its clarity and relevance to the construct of research study complexity. This approach permitted the researchers to focus on participant interpretations of individual survey items, relating their individual experiences to inform potential survey item revisions and to establish face and cognitive validity [13]. Interviews were audio recorded to ensure descriptive validity. One interviewer, with expertise in qualitative methods and instrument development, moderated all interviews. The participant, interviewer, and at least one other study team member were present during the interview session. Study team members took notes throughout the interview pertaining to item feedback, interpretation, and suggestion for revision. Immediately following each interview, the researchers reviewed all notes and discussed participant feedback to recognize and differentiate the interpretation presented by the participants and the researchers’ interpretations of the items. Iterative revisions occurred concurrently with each subsequent interview. Through constant comparison and principles of saturation, the team conducted interviews to further revise each item for clarity and relevance until there was consensus that saturation was achieved and no new information was emerging [14]. At this stage, the instrument was named the RCI.

### Phase 2. Pilot testing and initial psychometric analysis

We pilot tested the revised instrument to obtain initial item analyses and preliminary assessment of reliability [15]. We asked respondents to use the RCI to rate two preexisting protocols that were previously developed and implemented by a member of the study team. Because the targeted end user of the instrument may range from trainees to principal investigators, we purposively selected these protocols to ensure that they were not too complex thus allowing a universal understanding of study procedures to be scored. Johanson and Brooks recommend a minimum of 30 participants for initial scale development [16]. We recruited an additional convenience sample of clinical research staff through the team’s research network using a snowball technique and the same eligibility criteria as used in the face and validity testing [17]. We sent an email to potential participants explaining the project, its voluntary nature, and the research team’s contact information [18]. An electronic survey link was embedded in the email to permit participants to easily access the pilot instrument and two unique protocol exemplars [19]. The first protocol exemplar was a mixed-methods study designed to evaluate general cardiovascular risk among individuals with HIV. The second protocol exemplar was a randomized, placebo-controlled clinical trial to evaluate the efficacy of fish oil and a controlled diet to reduce triglyceride levels in HIV. Participants were asked to use the new version of the instrument that was based on the content and validity testing to score each protocol.

After 31 anonymous participants completed the pilot, a finalized dataset was established. The data were exported from REDCap to SPSS v.27 to perform initial psychometric analysis including item analysis and reliability statistics. Descriptive statistics, such as range, mean, and standard deviation, were computed for each item. Inter-item correlations (IIC) and the Cronbach’s alpha for the instrument were calculated [20]. Corrected item-total correlations were used to determine how each item correlates to other items in the instrument. A targeted range for IIC was 0.30–0.70 to prevent under- or over- correlation of the items on the instrument. Finally, a Fleiss’ Kappa statistic was used to assess inter-rater reliability. The Fleiss’ Kappa is a statistical measure for assessing reliability of agreement between more than three raters and is used for tools that have categorical response options [21]. Following pilot testing, our research team discussed the items to assess which items contribute to the overall scoring metric’s reliability and which items warrant further revision and/or removal [15].

## Results

### Phase 1

Participants completed the initial content validity testing using the electronic rating scale for each item and response option. Initial content validity indices indicated that 34 out of 100 collective items and scoring response options fell below the 0.8 reliability threshold and justified the need for revision during the cognitive interview phase. Seven people participated in a 1:1 interview to establish cognitive validity through iterative discussion and revision of each item and scoring response option.

At the conclusion of cognitive validity testing, all elements were retained and response options were revised to enhance clarity and relevancy for the following elements: selection of study instruments; physical equipment; budget preparation; consultant agreements; facilities or vendor agreements; hiring and job descriptions; access to target population; vulnerable populations; participant eligibility; incentives; IRB preparation; compliance reporting; expected AEs/safety risk; statistical analysis, and dissemination. Within each response option, criteria for each item were refined to scale complexity and included “Low (1 point),” “Moderate (2 points),” and “High (3 points).” The scale range of the instrument was 25 (low complexity) – 75 (high complexity).

### Phase 2

Thirty-three respondents returned the survey. We reviewed the datasets from the two individual protocol scores and used case-wise deletion to manage missing data. Specifically, responses with greater than 80% missing data for each protocol independently were removed. Subsequently, 31 total responses were received for each protocol. As shown in Table 3, most participants reported that they were female (74%) and White (72%). Over 80% of respondents had a master’s degree or higher. One-quarter of respondents reported their role as a principal investigator. Other roles included research coordinator (21.2%) and clinical research nurse (39%). There was a wide variability of research areas (e.g., genetics/genomics; oncology).

**Table 3.** Characteristics of respondents (phase 2 pilot testing)

	Count	Percentage	Valid percentage
**Highest degree**			
Associate’s degree	1	3.0	3.2
Bachelor’s degree	5	15.2	16.1
Master’s degree	15	45.5	48.4
Doctoral degree	10	30.3	32.3
**Research area of interest (select all that apply)**			
Health services research	8	24.2	24.2
Genetics/Genomics	16	48.5	48.5
Oncology	10	30.3	30.3
Informatics	1	3.0	3.0
Epidemiology	3	9.1	9.1
Public Health	5	15.2	15.2
Patient-centered outcomes research	14	42.4	42.4
Community-based participatory research	4	12.1	12.1
Other	3	9.1	9.1
**Race**			
Asian	2	6.1	6.1
White	24	72.7	72.7
Did not report	4	12.1	12.1
**Ethnicity**			
Hispanic	1	3.0	3.4
**Gender**			
Woman	23	69.7	74.2
Man	4	12.1	12.9
Did not Report	4	12.1	12.9
**Primary institutional affiliation**			
Academic/University	12	36.4	37.5
Research based hospital	8	24.2	25
Academic Medical Center	9	27.3	28.1
Pharmaceutical	1	3.0	3.1
**Role**			
Principal Investigator	8	24.2	24.2
Study/Research coordinator	7	21.2	21.2
Clinical Research Nurse	12	39.4	39.4
Grant/Finance	0	0	0
Student/Trainee*	2	6.1	6.1
**Mean Age (SD)**	49.31 (11.41)		

* Student/trainees had at least 5 years’ experience in clinical research and therefore met the eligibility criteria.

As shown in Table 4, item means and standard deviations, indicating item difficulty, ranged from 1.0 to 2.75 in Protocol exemplar 1 and 1.31 to 2.86 in Protocol exemplar 2. In Protocol 1, corrected item-total correlations, indicating item discrimination, ranged from 0.030 to .536. Fifteen items were under correlated to the other items in the scale. No items were over correlated. In Protocol 2, corrected item-total correlation ranged from 0.012 to .618. Ten items were under correlated while no items were found to be over correlated. Across both protocols, eight items were under correlated to the other items on the scale. They include facilities and vendor agreements (item 5), multiple PI agreements (item 6), access to target populations (item 9), vulnerable populations (item 10), participant eligibility (item 11), intervention administration (item 16), IRB preparation (item 20), and follow-up (item 23). Initial Cronbach’s alpha for the total scale was 0.586 in Protocol 1 and 0.764 in Protocol 2.

**Table 4.** Individual item analysis of the Research Complexity Index

	PROTOCOL 1 Cronbach’s Alpha = 0.586				PROTOCOL 2 Cronbach’s Alpha = 0.764			
	Mean	SD	Corrected item-total correlation	Cronbach’s Alpha if item deleted	Mean	SD	Corrected item-total correlation	Cronbach’s Alpha if item deleted
**1. Selection of study instruments**	2.75	0.44	0.423	0.550	2.52	0.574	0.317	0.756
**2. Physical equipment**	1.46	0.51	0.336	0.556	1.41	0.733	0.481	0.743
**3. Budget preparation**	1.14	0.36	0.053	0.588	1.48	0.738	0.427	0.747
**4. Consultant agreements**	1.32	0.55	0.263	0.565	1.83	0.602	0.587	0.739
**5. Facilities or vendor agreement**	1.75	0.44	0.060	0.589	1.93	0.371	0.206	0.761
**6. Multiple PI agreements**	1.35	0.68	0.030	0.601	1.41	0.628	0.185	0.764
**7. Hiring and job descriptions**	1.35	0.56	0.536	0.525	1.31	0.604	0.405	0.750
**8. Study arms**	1.35	0.73	0.507	0.516	1.93	0.371	0.121	0.764
**9. Access to target population**	1.78	0.74	0.149	0.583	1.97	0.731	− 0.012	0.779
**10. Vulnerable populations**	1.85	0.52	− 0.105	0.610	1.86	0.581	− 0.272	0.788
**11. Participant eligibility**	2.21	0.41	0.243	0.570	2.69	0.471	− 0.222	0.780
**12. Incentives**	2.67	0.61	− 0.365	0.650	2.55	0.827	0.068	0.776
**13. Informed consent**	1.82	0.48	0.113	0.583	2.41	0.568	0.520	0.744
**14. Randomization**	1.00	–	–	–	2.03	0.325	0.358	0.757
**15. Type of intervention**	1.07	0.26	0.104	0.584	2.03	0.421	0.307	0.757
**16. Intervention administration**	1.07	0.26	0.104	0.584	2.00	0.267	0.186	0.762
**17. Research team**	1.50	0.69	0.400	0.538	1.86	0.693	0.422	0.748
**18. Data collection (Procedures)**	2.03	0.69	0.530	0.515	2.17	0.759	0.527	0.739
**19. Data collection (Frequency)**	1.85	0.59	0.289	0.560	2.59	0.501	0.312	0.756
**20. Institutional Review Board approvals**	2.07	0.26	0.104	0.584	2.86	0.351	0.155	0.763
**21. Compliance reporting**	1.57	0.50	− 0.132	0.612	2.00	0.463	0.400	0.752
**22. Expected adverse events/safety**	1.10	0.31	0.210	0.575	1.45	0.572	0.592	0.739
**23. Follow-up**	1.14	0.36	0.134	0.581	1.83	0.658	0.189	0.764
**24. Statistical analysis**	1.46	0.58	0.261	0.564	1.90	0.900	0.618	0.729
**25. Dissemination**	1.39	0.69	0.125	0.586	1.52	0.688	0.533	0.740

* Item 14 had zero variance in protocol 1.

The range of total composite scores for Protocol 1 was 32 to 48 and for Protocol 2, 40-60, thus indicating that the second protocol was higher on the scale of complexity. Fleiss’ Kappa agreement for inter-rater reliability indicated fair agreement for both Protocol 1 (.338) and Protocol 2 (.277). Upon assessment of individual item ratings, there were four items (access to target population, research team, data collection procedures, and statistical analysis) that did not yield at least 60% agreement for a scoring tier. This indicates the poor performance of these items that may be driving the inter-rater reliability statistic lower. The final version of the RCI is displayed in Table 5.

**Table 5.** Research Complexity Index (final piloted version)

Research complexity instrument			
The purpose of this instrument is to scale the complexity of a research protocol.			
For each of the following elements, circle which level best fits the protocol.			
**Selection of study instruments (e.g., surveys, tools)**	1 instrument ***and*** Instruments validated in population.	2 to 3 instruments ***or*** Instruments valid but not validated in targeted population ***or*** At least 1 case report form; simple; 1-page form	4 or more instruments ***or*** Unknown validity ***or*** 2 or more case report forms that require multiple categorization; multiple pages
**Physical equipment**	Not applicable or Usual or standard care equipment (e.g. thermometer)	New to study team ***or*** Some learning required	Complex equipment in learning ***or*** Calibration needed
**Budget preparation/approvals**	2 or fewer authorizers	3-4 authorizers	5 or greater authorizers
**Consultant agreements**	Consultant Agreements (0)	Consultant Agreements that include different roles for each person (1-3)	Consultant Agreements that include different roles for each person (4 or more)
**Facilities or vendor agreement**	No facilities or vendors agreement needed	1-3 agreements required	4 or more facilities or vendors ***and/or*** new vendor/facility agreements need to be established
**Multiple principal investigators agreement**	Not applicable	1 multiple PI agreement needed	2 or more multiple PI agreement needed
**Hiring and job descriptions**	No new hires	1-3 new hires ***or*** At least 1 new job description needs to be developed	4 or more new hires ***or*** More than 1 new job description required
**Study arms**	1 arm ***or*** Data already available (e.g., secondary data analysis)	2 or 3 study arms	>4 study arms
**Access to target population (meets eligibility criteria)**	Most sites/locations routinely available	Targeted population accessible but new relationship needs to be established	Need to establish new relationships/access (e.g., media/ advertising) ***or*** Target population is uncommon/rare
**Vulnerable populations**	Not a vulnerable population	Targeted population included but does not require additional authorizations	Target participants includes vulnerable population that require additional authorizations (e.g., vented patient; adults without capacity; pediatrics)
**Participant eligibility screening**	No screening	Telephone/verbal screening ***or*** electronic health record review	In-person screening requires additional tests/EHR review to determine eligibility
**Incentives**	No incentives	One-time incentive	Incentives require multiple phases over the duration of the study period
**Informed consent process**	Written consent waived ***or*** Minimal Risk Consent ***or*** No participant representative needed	Written consent required but does not include complex explanation to participants ***or*** Simple trials with or without a placebo or pre/post study design ***or*** Consent requires language translation	Highly complex study to describe to participants that may require participant education (e.g. cross-over study, waitlist, blinding) ***or*** Studies involving multiple steps/ randomizations or intraoperative randomization ***or*** Participant surrogate needed
**Randomization**	One step; No randomization (i.e., observational study, cross-sectional survey)	Randomization without review of external department	Multiple steps/ randomizations ***or*** Intraoperative randomizations ***or*** Complex Central Pathology Review before randomization
**Type of intervention**	No intervention ***or*** Routine or standard of care (e.g., blood pressure; ECG)	Combined modality treatments ***or*** Simple inpatient treatments ***or*** Regimens with a defined # of cycles (sessions) ***or*** Cycles (sessions) of treatment are not defined. ***or*** Standard of care in addition to investigational agents or intervention	Outpatient/Ambulatory Intervention ***or*** Treatments with potential for increased toxicity (i.e. gene transfer, investigational bone marrow/ stem cell transplant, etc.) ***or*** Investigator/ site credentialing required ***or*** Extended administration of investigational agent or intervention, greater than 6 months
**Intervention administration**	No intervention ***or*** Routine or standard of care	Multiple points of administration within six months or less study duration	Greater than 6 months of intervention administration ***or*** DEA involvement for controlled substances ***or*** Intervention outside business hours (e.g., overnight stays/infusions) ***or*** Complex procedure or process to administer intervention, including short timeframe to administration (e.g. 24–48 hours from time of eligibility)
**Research team**	Standard clinical research team (internal to primary organization with no external collaboration)	Already established team with external institution collaboration	New research team with both internal and external disciplines/departments ***or*** Complex coordination outside primary team (e.g., across multiple departments or a large distance)
**Data collection procedure complexity**	Simple; Participant burden, less than one hour	Acquisition of existing data needed (e.g., EHR data mining) ***or*** Participant burden, per data collection point (study visit/session) one hour or more	Complex data collection procedures that require additional resources, personnel and/or facilities ***or*** Preliminary physiologic assessment or evaluation required prior to/during data collection ***or*** Specialist needed to collect samples (e.g., lumbar puncture) ***or*** Required refrigeration for climate-controlled samples
**Data collection frequency**	Data already available (e.g., retrospective data analysis) ***or*** One-time data collection point (e.g., cross sectional)	Prospective/longitudinal collection of data with at least 2 to 3 data collection points	Participant burden, per data collection point (study visit/session) requires in-patient admission ***or*** Multiple phases of data collection points
**Institutional Review Board approvals**	IRB prep (exempt)	IRB prep (expedited)	IRB prep (full board review)
**Compliance reporting (FDA, IRB, clinical trials registration, annual reports, regulatory reports)**	No external reporting required ***or*** No data sharing agreement needed external to organization.	Prospective submission of usual/ standard regulatory data ***or*** Standard NIH or funder progress reports; data integration plans ***or*** 1-3 Data sharing agreement(s) external to the primary institution	Complex prospective reporting to government/regulatory agency reporting ***or*** Data safety monitoring committee required ***or*** Auditors required ***or*** More than 3 data sharing agreement(s) external to the primary institution
**Expected adverse event/participant safety risk**	*None/Minimal Risk*	Moderate risk ***or*** Risk limited to 1 time over course of study	High Risk ***or*** Serious Adverse Events ***or*** Multiple episodes of moderate/high risk over course of study
**Protocol follow-up requirements**	No follow-up	Participant follow-up via phone call/virtual visits (in-person visit not required) ***or*** Simple coordination required for follow-up visits	Follow-up greater than 12 months ***or*** Complex coordination/frequent participant follow-up
**Statistical analysis**	Limited statistical analysis needed; Internal to study team	Analysis requires external statistical consultation once	Multiple consultations needed with biostatistician/bioinformationist to conduct advanced analysis beyond expertise of study team
**Dissemination and return of results**	Academic dissemination (Manuscript development/ Peer reviewed poster/podium) ***or*** No Return of Results to Study Participants	Academic dissemination (Manuscript development/ Peer reviewed poster/podium) ***and*** Public Media/Social media outreach (web based, you tube, twitter, LinkedIn) ***or*** Plan for Return of Results to Study Participants	All previous tiers ***and*** Implementation of study results into practice, policy or community adoption ***or*** Plan to obtain device/drug approval
**# boxes circled in each column**	________	_________	_______
**Multiply by**	**X 1 point**	**X 2 points**	**X 3 points**
**Total points each column**	________	________	________

Add all three columns to get final complexity score: ___________.

## Discussion

This project developed a new 25-item instrument to scale research study complexity. Following initial item development and psychometric pilot testing, the RCI demonstrates face and cognitive validity, but only fair inter-rater reliability. We found that some items were under correlated with each other despite participants indicating their critical nature when scaling complexity. This indicates that the conceptual foundation of the construct, study complexity, remains unclear. Conceptual analyses that refine the antecedents, dimensions, and consequences, of the construct of research study complexity should be explored concurrently with additional instrument revision and testing. Future testing should also include a larger sample to enable researchers to perform exploratory factor analyses. This may help form a more refined understanding of the conceptual underpinnings of the construct [22].

The findings of this study are aligned with existing literature that notes the challenges of scaling research complexity. Project difficulty across all fields (e.g., engineering) is often defined as how hard it is to achieve goals and objectives [23]. An empirical measure is helpful to quantify operational performance, allocate resources and personnel, and establish metrics for project or individual researcher success [24]. In academic medical institutions, researchers and academic leadership have noted the importance of recognizing resources, finances, and the establishment of guidelines and measurement systems to scale faculty effort in research [25]. Some argue that, in lieu of determining effort by one’s level of grant support, transparent metrics are needed to help researchers distinguish the complexity of their activities and responsibilities [26]. The RCI proposed by this study may better capture study complexity and allow researchers to better demonstrate the time, effort, and allocated resources regardless of study design or funding.

Similar to the goals of the original NCI Trial Complexity Model [8], the proposed RCI may also be useful for estimating funding or resources required by the study’s most time-consuming tasks. Further, institutional allocation of resources is sometimes based on the level of acquired funding, and not necessarily informed by study design, proposed workload, or researcher experience. Yet, the experience level of investigators should be taken into consideration when scaling complexity. For example, a principal investigator with decades of experience conducting clinical trials may consider tasks less complex as compared to an early-stage investigator. The goal of any instrument used to measure research complexity should be to inform organizations how to best optimize research efficiency and cost-effectiveness through early and accurate evaluation of researcher needs. Across fields, there is some evidence that the three determinants of research efficiency include seniority, public funding, and institutional reputation [27]. Yet, it is recommended that institutions formulate strategies to better measure and promote operational and performance improvement [28,29]. As part of the ongoing development of this present instrument, we recommend future validity and reliability testing across settings with researchers who have varying levels of experience. Subsequently, we may grasp a better understanding of the stewardship of research resources (i.e., time, staff, budgets) needed by trainees, junior scientists, or senior faculty across all study designs [30].

### Limitations

This research project has limitations that should be considered prior to widespread adoption of the new instrument. First, we acknowledge that not all researchers have the same experience with all study design types (e.g. clinical trials) thus presenting potential variability of responses. Varying institutional-specific resource access may also alter the complexity of a protocol. However, since the objective of this study was to create a more universal instrument when measuring complexity across researcher- and institution-types we believe the initial piloted version serves as a sufficient prototype prior to additional testing. Future research may include homogenous clusters of researchers based on level of experience and familiarity with specific study designs. Second, while effective in targeting experienced clinical research staff, the purposive sampling strategy may not have encompassed all categories of staff involved in clinical research. We state that variation in research fields may present challenges using a universal scale that captures study complexity. However, our design built in variations with protocol exemplars and evaluated the instrument with participants of various levels of experience to allow a more rigorous analysis. The authors recognize that diversity, equity, and inclusion (DEI) is an important variable to assess in a study; however, this instrument may not capture a study’s DEI complexity. Additionally, the lack of a user manual for the study participants was another limitation that may have impacted the usability and effectiveness of the Research Complexity Index. Our findings suggest that further refinement of these terms, a user manual, and additional training may be necessary for study teams to effectively use the HCRI instrument. These will be included in the next phase of instrument development.

## Conclusion

This paper presents the development and initial psychometric properties of the RCI, which demonstrates early validity and reliability. While this instrument is still in its initial stages, the potential to assist in study planning, resource allocation, and personnel management is valuable. Further construct refinement and additional psychometric testing, including factor analyses, will allow for the evaluation of construct validity.
